# A Rare Case of Pancreaticopleural Fistula That Presented as a Recurrent Pleural Effusion at a Regional Hospital in Guyana

**DOI:** 10.7759/cureus.61357

**Published:** 2024-05-30

**Authors:** Ravinauth Thakoordeen, Mahindra Rampersaud, Tameshwar K Algu

**Affiliations:** 1 General Surgery, New Amsterdam Regional Hospital, Berbice, GUY; 2 Department of Surgery, New Amsterdam Regional Hospital, Berbice, GUY

**Keywords:** pancreatitis, pancreaticojejunostomy, recurrent pleural effusion, pancreaticopleural fistula, hepatic-bilio-pancreatic surgery

## Abstract

Pancreaticopleural fistula is a rare complication of pancreatitis. We present a rare case of pancreaticopleural fistula in a 43-year-old alcoholic male. He presented with recurrent episodes of left pleural effusion that were managed with aspiration and chest tube placement. An MRI of the chest and upper abdomen revealed a pancreaticopleural fistula. The patient underwent distal pancreatectomy with splenectomy and Roux-en-Y pancreaticojejunostomy. The surgical approach was our first-line management due to the unavailability of octreotide and endoscopic retrograde cholangiopancreatography. His recovery was complicated by an empyema that was managed by tube thoracostomy and IV antibiotics. There was no issue detected at his 3-month follow-up clinic visit.

## Introduction

Pancreaticopleural fistula is a rare clinical pathology that results from disruption of the main pancreatic duct. It can be seen in patients with pancreatitis or patients following traumatic and surgical disruption of the pancreatic duct [1-3]. Pancreaticopleural fistula usually forms as a result of a leak from a ruptured pseudocyst, or as a leak from an incompletely formed pseudocyst, or rarely from a direct pancreatic duct leak. The fistulous tract can pass directly through the diaphragm or through the aortic or esophageal openings in the diaphragm. This form of internal pancreatic fistula is characterized by massive pleural effusion. Treatment options for pancreaticopleural fistula include (i) octreotide and thoracocentesis/thoracostomy, (ii) endoscopic retrograde cholangiopancreatography (ERCP) and stenting together with octreotide, and (iii) surgical management that includes pancreatic resection and enteropancreatic drainage [1-5]. ERCP is expensive and not readily available in developing, resource-poor nations. Complete resolution occurs in 80 to 90% of cases treated surgically. New Amsterdam Regional Hospital is a resource-poor regional hospital in Guyana that serves a population of approximately 159,000. ERCP is not available in the public healthcare system in the country.

## Case presentation

**Figure 1:**
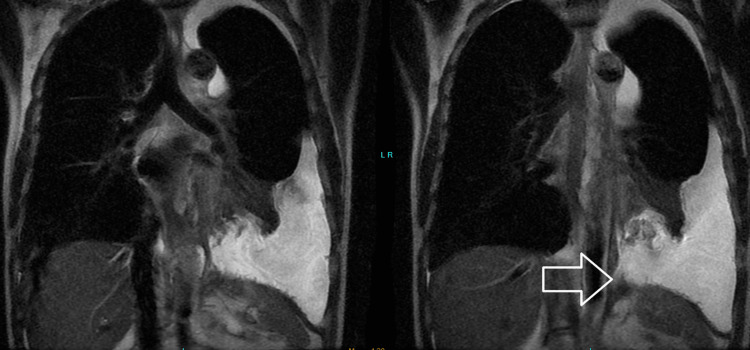
MRI of the chest demonstrating pancreaticopleural fistula (arrow pointing out the fistula tract).

This is the case of a 43-year-old alcoholic male, who presented with a history of chest pain, back pain, and dyspnea. He had no abdominal complaints. His erect chest X-ray revealed a large left pleural effusion. The patient was admitted under the care of the internal medicine physicians of the New Amsterdam Hospital. A thoracocentesis was performed where approximately a liter of cloudy fluid was drained. A chest tube was passed four days subsequently as he developed a recurrent effusion. After removal of the tube, the patient had a recurrent pleural effusion within a week and another chest tube had to be passed. After removal of the tube on the second instance an MRI of the chest and upper abdomen was requested. This revealed a left pleural effusion and a pancreaticopleural fistula (Figure 1).

**Figure 2:**
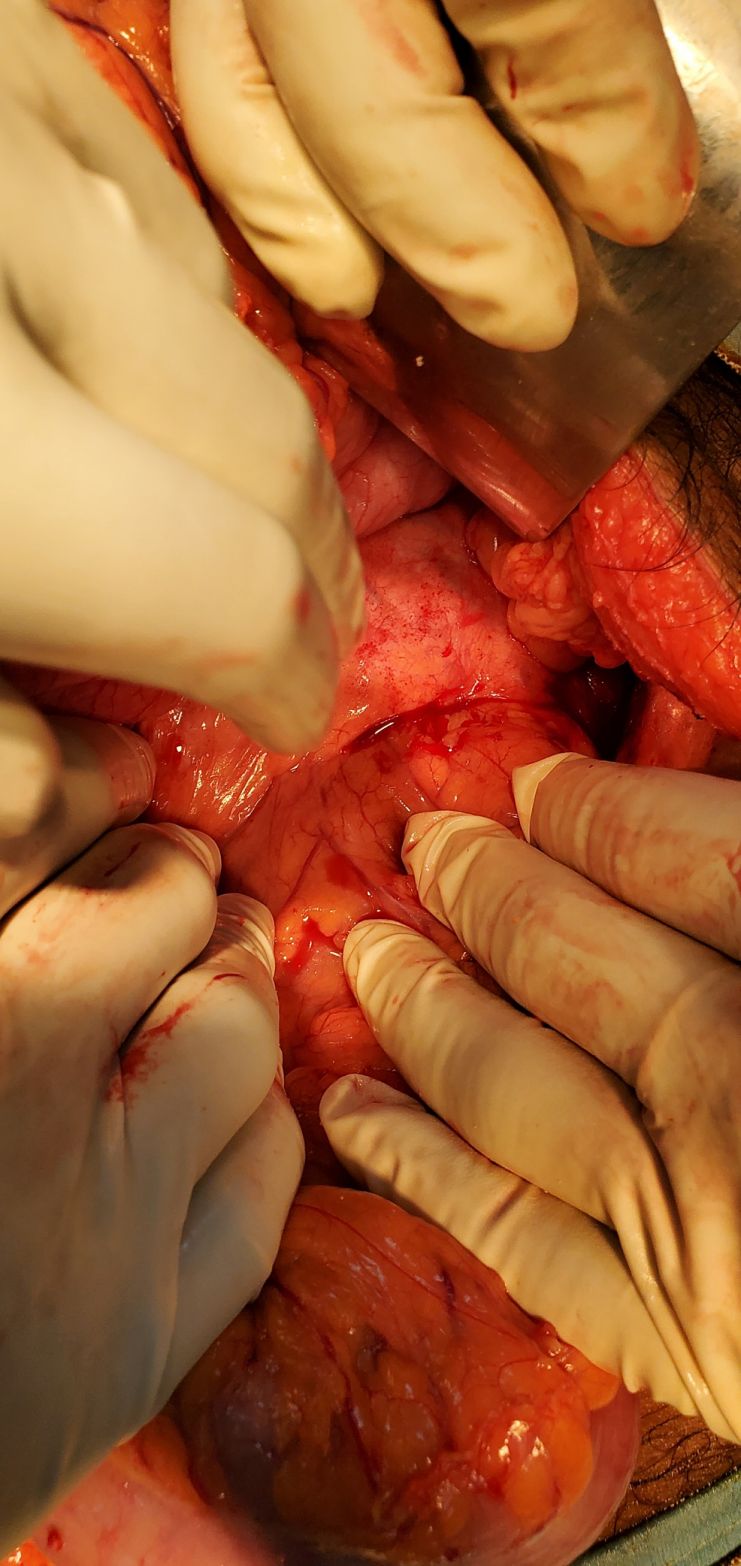
Pancreaticopleural fistula between distal pancreas and esophageal hiatus of the diaphragm.

**Figure 3:**
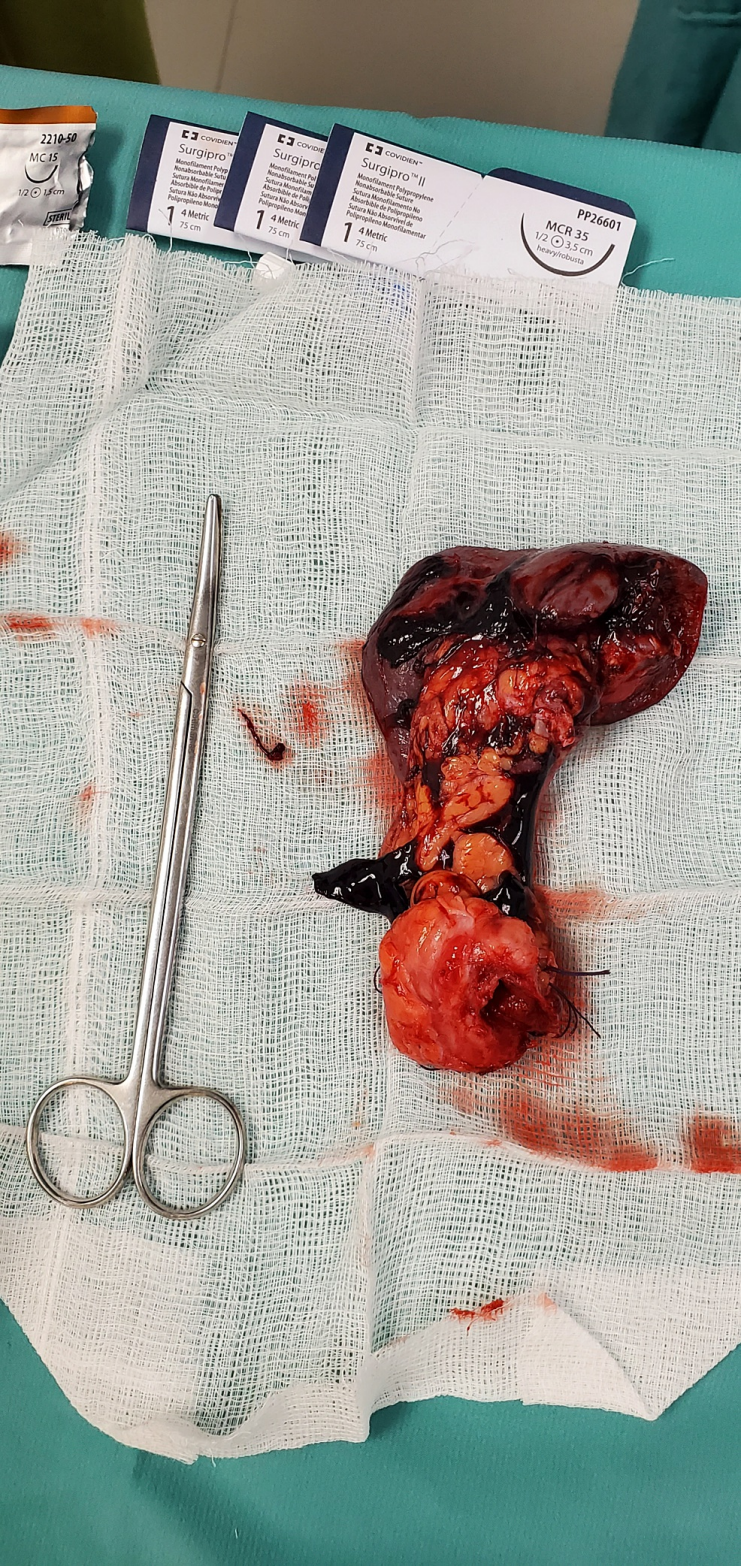
Specimen of distal pancreas and spleen.

**Figure 4:**
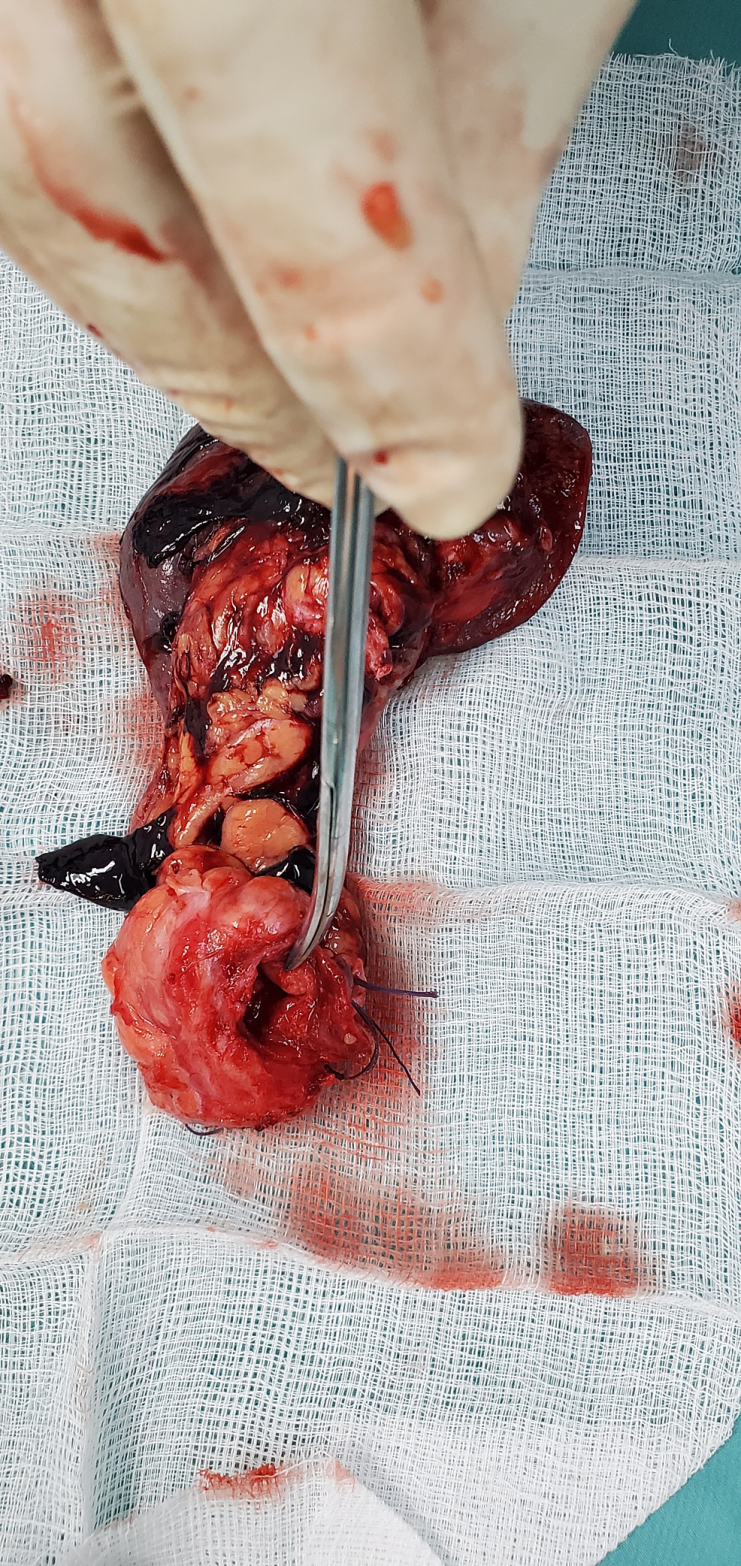
Specimen of distal pancreas with dilated pancreatic duct.

**Figure 5:**
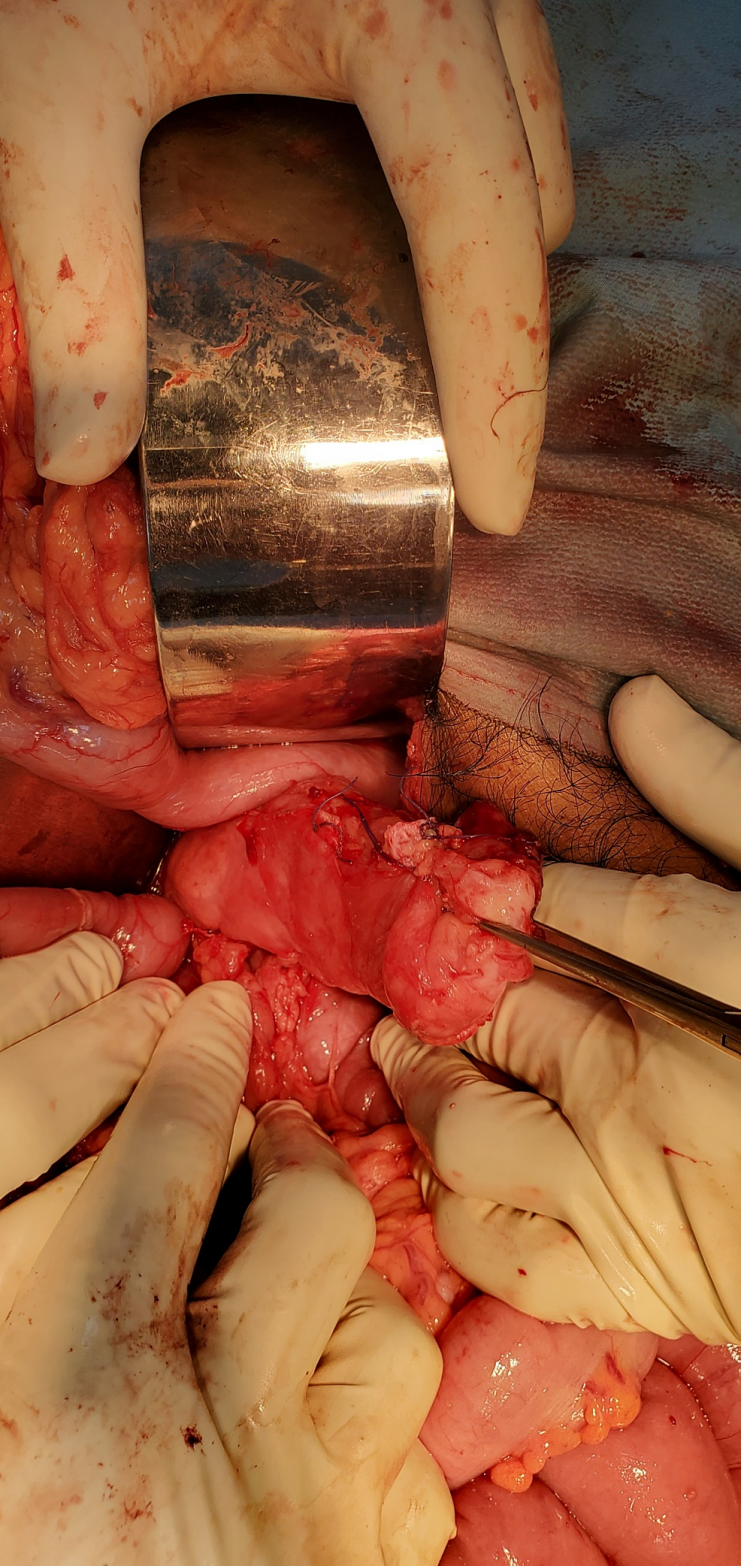
Body of pancreas after distal pancreatectomy.

**Figure 6:**
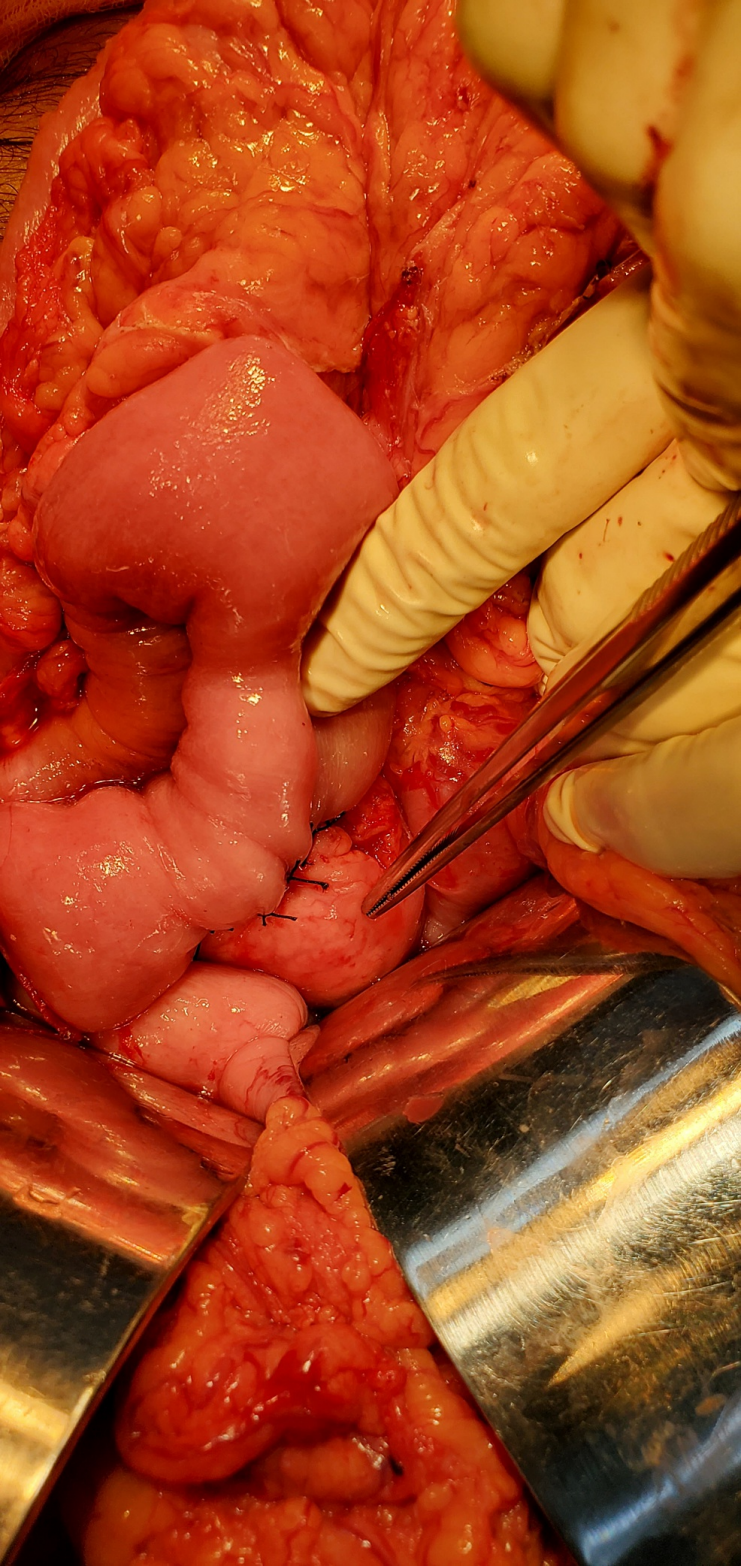
Completed Roux-en-Y pancreaticojejunostomy.

He was admitted under the general surgery service and after informed consent, he underwent a laparotomy with findings of a pancreaticopleural fistula between the distal pancreas and esophageal hiatus of the diaphragm (Figure 2). Distal pancreatectomy with splenectomy and Roux-en-Y pancreaticojejunostomy were performed (Figures 3-6).

His postoperative recovery was complicated by a left empyema that was managed with tube thoracostomy and intravenous antibiotics. His 3 months post-surgery follow-up did not reveal any issues with his recovery.

## Discussion

This patient, a known alcoholic, may have developed a pancreaticopleural fistula as a complication of pancreatitis. This type of fistula is seen in 3 to 7% of patients with pancreatitis [1]. Pancreaticopleural fistula was not suspected in this patient initially and his diagnosis was based on imaging via MRI of the chest and upper abdomen. This patient did not have any abdominal issues which is a classic feature in patients with pancreaticopleural fistula.

Although surgery is generally a second-line management which is usually done after medical management and ERCP fails, our institution did not have octreotide and ERCP is not available in the public healthcare system therefore surgery in the form of pancreaticojejunostomy was done. We believe that the surgical approach was safe with a success rate of approximately 90% vs 30 to 60% in patients being managed conservatively. Early surgical intervention also has the advantage of reducing the duration of therapy for the resolution of the fistula by 50% [1,5].

## Conclusions

Diagnosing a pancreaticopleural fistula requires a high index of suspicion. Patients with recurrent pleural effusion should have advanced imaging investigations to determine the cause. Early surgical intervention is an accepted safe modality of treatment in resource-poor hospitals where ERCP is not available. Early surgical intervention in this case had the advantage of reducing the time for resolution of the pancreaticopleural fistula.
